# Pcsk9 is associated with severity of coronary artery lesions in male patients with premature myocardial infarction

**DOI:** 10.1186/s12944-021-01478-w

**Published:** 2021-05-27

**Authors:** Jing Gao, Ya-Nan Yang, Zhuang Cui, Si-Yuan Feng, Jing Ma, Chang-Ping Li, Yin Liu

**Affiliations:** 1 Chest Hospital, Tianjin University, No.92 Weijin Road Nankai District, Tianjin, 300072 P.R. China; 2 Thoracic Clinical College, Tianjin Medical University, No.22 Qi xiang tai Road, Heping District, Tianjin, 300070 P.R. China; 3 Cardiovascular Institute, Tianjin Chest Hospital, No.261 Tai erzhuang Road, Jinnan District, Tianjin, 300222 P.R. China; 4 Tianjin Medical University, No.22 Qi xiangtai Road, Heping District, Tianjin, 300070 P.R. China; 5 Department of Cardiology, Tianjin Chest Hospital, No.261 Tai erzhuang Road, Jinnan District, Tianjin, 300222 P.R. China

**Keywords:** Coronary angiography, Coronary artery disease, Premature myocardial infarction, Proprotein convertase subtilisin/kexin type 9

## Abstract

**Background:**

Proprotein convertase subtilisin/kexin type 9 (Pcsk9) correlated with incidence and prognosis of coronary heart disease. However, it is unclear whether Pcsk9 contributed to coronary artery lesion severity in patients with premature myocardial infarction (PMI). The present study investigated associations between Pcsk9 and coronary artery lesion severity in PMI patients who underwent coronary angiography (CAG).

**Methods:**

This prospective cohort study included young men (age ≤ 45 years, *n* = 332) with acute MI who underwent CAG between January 2017 and July 2019. Serum Pcsk9 levels and clinical characteristics were evaluated. SYNTAX scores (SYNergy between percutaneous coronary intervention with [paclitaxel-eluting] TAXUS stent and cardiac surgery) were calculated to quantify coronary artery lesions.

**Results:**

Serum Pcsk9 levels were positively associated with SYNTAX scores (*r* = 0.173, *P* < 0.05). The diagnostic cutoff value of PSCK9 level was 122.9 ng/mL, yielding an area under the curve (AUC) of 0.63, sensitivity 81%, and specificity 40%. Serum Pcsk9, LDL-C, Apob, NT-proBnp, CK level, and diabetes history were independent predictors of high SYNTAX scores (*P* < 0.05). After stratifying by serum LDL-C level (cutoff = 2.6 mmol/L), medium-high Pcsk9 levels had increased risk of high SYNTAX scores in patients with high LDL-C (*P* < 0.05), and higher serum Pcsk9 levels had increased risk of major adverse cardiac events (MACE) after adjusting for confounding factors (*P* < 0.05).

**Conclusion:**

Serum Pcsk9 levels correlates with severity of coronary artery lesion in PMI patients and may serve as a biomarker for severity of coronary artery stenosis in this patient population, which may contribute to risk stratification.

**Supplementary Information:**

The online version contains supplementary material available at 10.1186/s12944-021-01478-w.

## Background

The high incidence and mortality of myocardial infarction (MI) pose a threat to public health. More than 600,000 people have experienced myocardial infarction, which causes 180,000 deaths each year in China [1, 2]. In recent years, the incidence of premature myocardial infarction (PMI) has gradually increased [3]. PMI is generally defined as MI for men ≤55 years and women ≤65 years [4]. Arora et al. [5] found that the proportion of young patients hospitalized for acute MI increased significantly from 27 to 32% between 1995 and 2014, and 1-year mortality of younger patients with AMI was nearly 10%. Genetics play a critical role in the progression of PMI, with a heritability of 63% [6]. Additional evidence reveals that proprotein convertase subtilisin/kexin type 9 (Pcsk9) relates to cholesterol homeostasis, is a risk factor for PMI [7–9]. As a relatively new PMI risk factor, Pcsk9 has received widespread attention for elevating plasma low-density lipoprotein cholesterol levels due to promote degradation of LDL receptor in the liver [10, 11]. Pcsk9 is related to incidence and prognosis of coronary artery disease (CAD) and with lipid or non-lipid cardiovascular risk factors [12–14]. The increasing clinical application of Pcsk9 inhibitors is considered to be an effective treatment to reduce major adverse cardiovascular events (MACE), MI and stroke, which has not a significant safety concerns [15, 16].

Various scoring systems are used to assess the extent of CAD, including SYNergy between percutaneous coronary intervention with TAXus and cardiac surgery (SYNTAX) [17], and Gensini and Jeopardy scores [18]. According to the 2018 ESC/EACTS guidelines [19], SYNTAX score was applied to assess the coronary artery lesion. Results of multiple studies indicate that Pcsk9 may be associated with CAD severity and plaque load [20–24]. A few studies have determined association of Pcsk9 levels with the extent of CAD [22, 25], although this relationship remains controversial. To date, no study has investigated correlations between Pcsk9 and CAD severity in PMI patients. Therefore, this study aimed to determine association of Pcsk9 with CAD severity in patients with PMI.

## Materials and methods

### Study population

This prospective study included 332 patients (aged ≤45 years) diagnosed with acute MI according to the Fourth Universal Definition of Myocardial Infarction [26] or the International Classification of Diseases, 10th revision, Clinical Modification (ICD-10-CM) codes (ICD-10-CM I21). Patients were recruited from the cardiac/coronary care unit of Tianjin Chest Hospital, China, from January 2017 to July 2019. Inclusion criteria were: patients aged ≥18 years presenting within 1 day (preferably within 12 h) after pain onset with a principle diagnosis of non-ST elevation myocardial infarction (NSTEMI), or ST-elevation myocardial infarction (STEMI). Enrolled patients had at least one of the following criteria: (i) ECG ischemic changes such as persistent or dynamic ST-segment deviation, T-waves inversion, new left bundle branch block; (ii) evidence of positive conventional or high-sensitive troponin by local laboratory reference values with a rise and/or fall in enzyme levels; (iii) patients who underwent coronary angiography (CAG), had one or more coronary artery stenosis (stenosis ≥50%), and received percutaneous coronary intervention (PCI) within 12 h. Exclusion criteria were: subjects with prior coronary artery bypass graft (CABG) surgery, female, antihyperlipidemic agents within 3 months, infectious, systemic inflammatory disease, or severe hepatic and/or renal failure.

### Ethical considerations

The study protocol was approved by the Ethics Committee of Tianjin Chest Hospital (No. 2017KY-007-01) and all included patients provided signed informed consent prior to study participation. All procedures performed were in accordance with the ethical standards of the Helsinki Declaration and its later amendments, or comparable ethical standards.

### Measurement of clinical and laboratory parameters

A data checklist included demographic information, medical history (hypertension, diabetes, familial history of premature coronary artery disease, and history of tobacco smoking), body mass index (BMI), blood pressure (BP), left ventricular ejection fraction (LVEF) based on echocardiography. Conventional risk factors for CAD, including hypertension [27], diabetes [28], and smoking status [29] were evaluated. Laboratory parameters were measured by standard clinical laboratory techniques. Blood samples were taken after an overnight fasting (≥ 8 h) on the day of admission to determine serum levels of apolipoprotein B (ApoB), total cholesterol (TC), low-density lipoprotein cholesterol (LDL-C), high-density lipoprotein cholesterol (HDL-C), triglycerides (TG), alanine transaminase (ALT), creatinine, high-sensitivity C-reactive protein (hs-Crp) and N-terminal pro-B type natriuretic peptide (NT-proBnp).

### Measurement of Pcsk9

After overnight fasting (≥ 8 h) on the day of admission, blood samples were collected in tubes with EDTA anticoagulant (ethylene diamine tetra-acetic acid) in the morning and centrifuged at 3000 rpm at 4 °C for 10 min to obtain serum. All samples were stored at − 80 °C until analysis. Serum Pcsk9 levels were analyzed by enzyme linked immunosorbent assay (ELISA) (R&D Systems, Minneapolis, MN, USA) and samples were diluted 1:1 in dilution buffer prior to assay.

### SYNTAX scores

Angiographic images of all patients were evaluated and SYNTAX scores (SS) were calculated. SS is a tool for grading complexity and severity of CAD based on angiography. It is an anatomical scoring system that quantitatively characterizes coronary arteries based on location, function, complexity and number of obstructions, evaluating vessels with diameters greater than 1.5 mm and stenosis greater than 50%. Each segment of the coronary artery has a weighting factor determined by lesion location and severity. A SYNTAX calculator (www.syntaxscore.com) is used to synthesize lesion features to obtain final scores. The SYNTAX scores were assessed by three experienced cardiologists who were blinded to Pcsk9 concentrations. If disagreement occurs, final decisions are determined by consensus.

### Patient follow-up and main outcomes

According to CAG, all patients received appropriate treatment modalities (including PCI) from their cardiologists. Patients were followed prospectively at 6, 12, 24, 36 months after diagnosis. The primary outcome included cardiac death, non-fatal stroke, non-fatal recurrent myocardial infarction, post-discharge revascularization (PCI/CABG), and re-hospitalization for heart failure. Cardiac death was confirmed by death from cardiac causes, including sudden cardiac death, congestive HF, acute MI, severe arrhythmia, stroke, or other structural/functional cardiac diseases. Stroke was defined as an acute cerebral infarction on the basis of imaging results or typical symptoms. MI was diagnosed based on a comprehensive evaluation combining equivalent symptom complex or chest pain, changes in cardiac enzyme levels, and electrocardiogram findings.

### Statistical analysis

Statistical analysis was carried out using SPSS 25 software (SPSS Inc., Chicago, Illinois, USA). Before applying the corresponding test, the normality of the distribution in variables have been verified. The normality of all continuous variables were verified by Kolmogorov-Smirnov test. Tertiles were used for the scoring system (SS ≤ 12,> 12 < SS ≤ 21.5, SS > 21.5). The results were expressed as percentage, mean (±SD) or median (IQR). Comparison of continuous variables between groups were performed by One-way analysis of variance (ANOVA) or Kruskal-Wallis tests. Comparison of categorical variables between the group were performed by Chi-square analysis. Spearman correlation test was used to determine relationships between targeted parameters. Multivariate logistic regression analysis was used to calculate odds ratios (ORs) of high SYNTAX scores (> 21.5) for patients with tertile 2 (T2) and tertile 3 (T3) versus patients with tertile 1 (T1) grouped according to Pcsk9 levels. The model was also adjusted for various factors. Significant parameters in univariate analysis (*P*-value< 0.05) were entered into multivariate linear regression analysis to determine the predictors of SYNTAX scores. Univariate and multivariate Cox regression analyses were performed to calculate hazard ratios (HRs) of MACE. A Kaplan-Meier curve was graphed for MACE-free survival times of patients by T1, T2, and T3 grouped according to Pcsk9 levels. All tests were two-sided and *P* < 0.05 was considered statistically significant.

## Results

### Predictive factors for SYNTAX scores

Angiography results of PMI patients showed that145 patients (43.70%) had one vessel lesion, 100 patients (30.10%) had two vessel lesions, and 87 patients (26.20%) had three vessel lesions. PMI patients were grouped into 3 subgroups by SYNTAX scores (Table 1). All patients did not have familial hypercholesterolemia, prior statin use, and prior ezetimibe use, while34 patients (10.30%) had familial premature coronary artery disease (PCAD), 44 patients (13.30%) had history of beta-blocker use, 34 patients (13.30%) had history of CCB use, 32 patients (9.70%) had history of ARB/ACEI use. However, proportions of familial PCAD, beta-blocker use, CCB use, and ARB/ACEI use were not significant difference between the three groups (all *P* > 0.05). Particularly, LVEF, diabetes history, and serum LDL-C, ApoB, glucose, and Pcsk9 levels were significantly different between these subgroups (all *P* < 0.05). Proportions of diabetes history, serum LDL-C, ApoB, glucose and Pcsk9 levels were highest in the SS > 21.5 group, while the proportion of LVEF was lowest in the SS > 21.5 group. Predictors of SYNTAX scores were identified by univariate linear regression analysis (*P* < 0.05, Table 2). Subsequently, multivariate linear regression analysis indicated that serum Pcsk9, Apob, NT-proBnp and CK levels and diabetes history were positively associated with high SYNTAX scores (all *P* < 0.05).

**Table 1:** Baseline clinical, demographic, biochemical and angiographic features of patients in different SYNTAX score (SS) groups

Variables	Total (***N*** = 332)	SS ≤ 12 (***n*** = 114)	12 < SS ≤ 21.5 (***n*** = 113)	SS > 21.5 (***n*** = 105)	***P-***value
**Age** (years)	39.30 (4.50)	39.30 (4.70)	39.60 (4.70)	39.10 (3.90)	0.57
**Body mass index**, kg/m2	25.00 (2.51)	25.28 (2.70)	24.64 (2.30)	25.06 (2.51)	0.46
**SBP**, mmHg	132.50 (23.20)	132.80 (21.40)	133.20 (24.30)	131.30 (24.20)	0.96
**DBP**, mmHg	81.0 (14.7)	80.2 (14.5)	84.1 (15.1)	78.5 (14.2)	0.07
**Past history**					
Hypertension	148 (44.60)	44 (39.00)	49 (43.40)	55 (52.10)	0.26
Diabetes	46 (13.80)	8 (7.00)	12 (10.60)	27 (25.70)	< 0.01*
Familial hypercholesterolemia	0 (0)	0 (0)	0 (0)	0 (0)	**–**
Familial PCAD	34 (10.30)	16 (14.00)	9 (8.00)	9 (8.60)	0.36
Smoking	256 (77.10)	89 (78.10)	83 (73.50)	84 (80.00)	0.63
**MI type**					
STEMI	289 (87.10)	95 (83.30)	96 (85.00)	98 (93.30)	0.18
NSTEMI	43 (12.90)	19 (16.70)	17 (15.00)	7 (6.70)	
**Biochemistry**					
White blood cells, 10^9^/L	11.45 (3.12)	11.23 (3.29)	11.20 (2.56)	11.95 (3.46)	0.21
Urea, mmol/L	4.69 (1.64)	4.48 (2.34)	4.55 (1.70)	5.05 (1.89)	0.05
Lp(a), nmol/L	21.15 (51.38)	19.00 (33.65)	18.05 (51.58)	24.95 (53.63)	0.28
TC, mmol/L	5.01 (1.12)	4.84 (0.98)	4.98 (1.19)	5.22 (1.16)	0.11
LDL-C, mmol/L	3.35 (1.04)	3.18 (0.92)	3.26 (0.98)	3.63 (1.17)	0.02*
VLDL-C, mmol/L	0.49 (0.48)	0.55 (0.53)	0.48 (0.48)	0.49 (0.43)	0.87
HDL-C, mmol/L	0.96 (0.31)	0.98 (0.42)	0.92 (0.25)	0.97 (0.20)	0.25
Triglycerides, mmol/L	2.11 (1.49)	2.12 (1.35)	2.11 (1.53)	2.09 (1.42)	0.99
Apob, g/L	1.20 (0.30)	1.15 (0.27)	1.17 (0.29)	1.29 (0.32)	< 0.01*
ALT, U/L	54.10 (41.55)	48.75 (47.45)	55.55 (39.98)	54.5 (41.78)	0.97
Glucose, mmol/L	5.62 (2.93)	5.52 (1.88)	5.51 (2.35)	6.52 (4.06)	0.04*
Creatinine, μmol/L	79.54 (19.74)	77.56 (12.30)	76.96 (16.82)	84.44 (27.16)	0.09
**Coronary heart disease**					
LVEF, %	49.80 (7.70)	51.30 (7.10)	50.40 (7.80)	47.60 (7.70)	< 0.01*
Creatine kinase, U/L	1649.50 (2451.00)	1324.00 (1903.00)	1744.00 (2332.80)	1950.00 (2674.00)	0.18
CK-MB, U/L	125.5 (189.8)	111.5 (184.0)	121.0 (174.0)	162.0 (249.0)	0.33
c-Tnt, ng/mL	3.57 (5.14)	2.61 (5.38)	3.47 (4.62)	4.01 (5.14)	0.15
hs-Crp, mg/L	6.18 (13.01)	6.70 (15.04)	5.55 (11.68)	5.92 (13.06)	0.88
NT-proBnp, pg/mL	446.0 (832.4)	407.6 (809.8)	437.5 (789.5)	528.1 (905.2)	0.29
**Prior drug use**					
Statin	0 (0)	0 (0)	0 (0)	0 (0)	–
Ezetimibe	0 (0)	0 (0)	0 (0)	0 (0)	–
Beta-blocker	44 (13.30)	16 (14.00)	14 (12.40)	14 (13.30)	0.22
CCB	34 (10.20)	11 (9.70)	14 (12.40)	9 (8.60)	0.45
ARB/ACEI	32 (9.70)	9 (7.90)	11 (9.70)	12 (11.40)	0.31
**Biomarker**					
Pcsk9, ng/mL	175.66 (92.9)	160.72 (71.39)	170.21 (112.64)	197.95 (87.38)	< 0.01*

Variables are presented as mean (SD), median (IQR), or n (%), *P-*values were derived from one-way analysis of variance, the Kruskal-Wallis test or χ2 test. * *P* < 0.05

*SBP* systolic blood pressure, *DBP* diastolic blood pressure, *PCAD* premature coronary artery disease, *Lp(a)* lipoprotein a, *TC* total cholesterol, *LDL-C* low density lipoprotein cholesterol, *Apob* apolipoprotein B, *HDL-C* high density lipoprotein cholesterol, *ALT* alanine transaminase, *LVEF* left ventricular ejection fraction, *CK-MB* creatine kinase MB, *hs-Crp* high-sensitivity C-reactive protein, *c-Tnt* cardiac Troponin T, *NT-proBnp* N-terminal pro-B type natriuretic peptide, *CCB* calcium channel blocker, *ARB/ACEI* angiotensin receptor blocker/angiotensin converting enzyme inhibitor, *Pcsk9* proprotein convertase subtilisin/kexin type 9. * *P* < 0.05

**Table 2:** Univariate and multivariate linear regression analysis of SYNTAX score and predictors

Predictors	Univariate		Multivariate	
	***β***	***P-***value	***β***	***P-***value
**Pcsk9**, ng/mL	0.017	0.010*	0.013	0.027*
**Apob**, g/L	5.778	0.004*	4.559	0.020*
**Glucose**, mmol/L	0.393	0.028*	–	–
**Creatine kinase**, μmol/L	0.001	< 0.001*	0.001	0.001*
**c-Tnt**, ng/mL	0.511	0.005*	–	–
**NT-proBnp**, pg/mL	0.001	0.021*	0.002	0.011*
**LVEF**, %	−0.280	< 0.001*	–	–
**LDL-C**, mmol/L	1.516	0.008*	–	–
**Diabetes**	5.091	0.003*	3.869	0.025*

*Pcsk9* proprotein convertase subtilisin/kexin type 9, *Apob* apolipoprotein B, *c-Tnt* cardiac Troponin T, *NT-proBnp* N-terminal pro-B type natriuretic peptide, *LVEF* left ventricular ejection fraction, *LDL-C* low density lipoprotein cholesterol. *P < 0.05

### Pcsk9levels and CAD severity

**Fig. 1:**
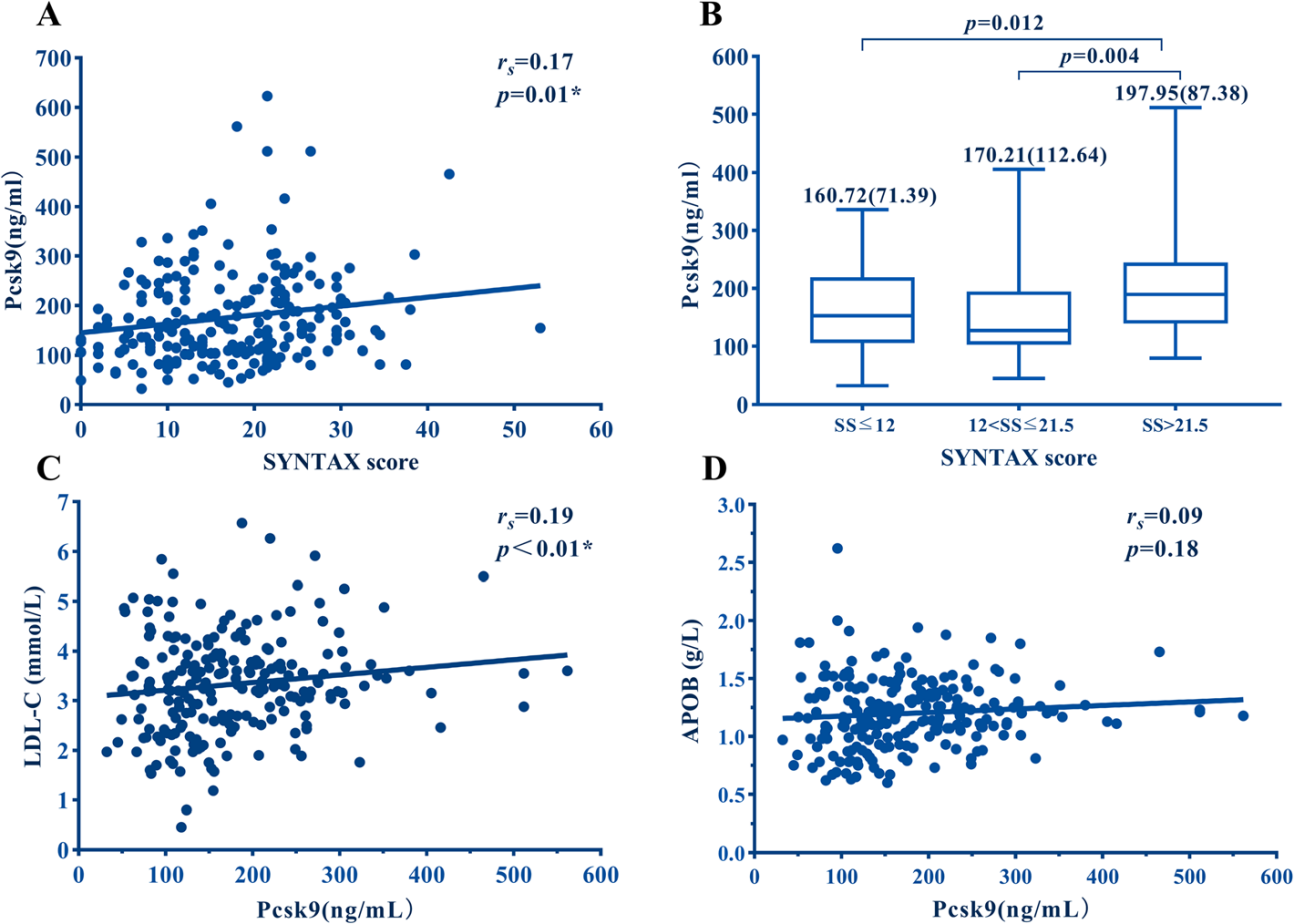
Association of Pcsk9 with coronary severity. **a** Pearson Correlation analysis represents the relationship between Pcsk9 and SYNTAX score. **b** Pcsk9 levels in relation to coronary severity. Coronary severity was assessed by SYNTAX scoring system and patients were divided into 3 groups according to SYNTAX scores. Pcsk9 levels were all significantly associated with coronary severity assessed by the SYNTAX scoring system (all *P* < 0.05). **c-d** Pearson correlation analysis represents the relationship between Pcsk9 with LDL and Apob

**Fig. 2:**
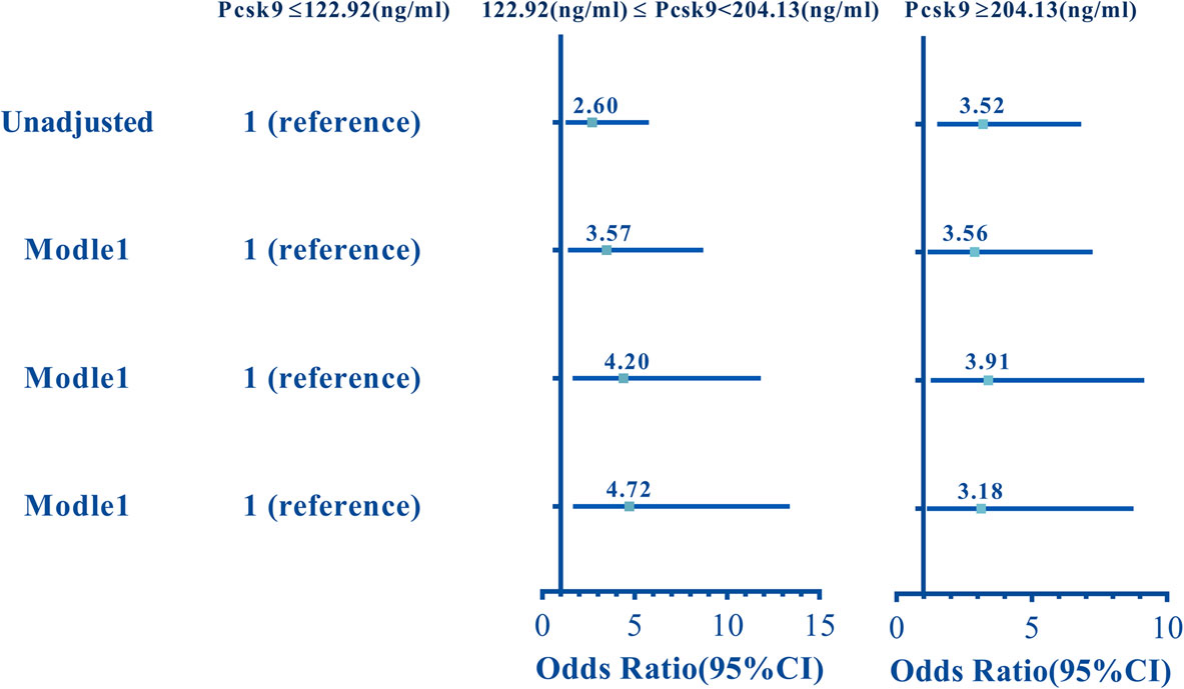
Risk of high SYNTAX scores in patients with different Pcsk9tertiles. Logistic regression Model 1 was adjusted for age and body mass index (BMI). Logistic regression Model 2 was adjusted for age, BMI, familial history of PCAD, smoking, hypertension, and diabetes. Logistic regression Model 3 was adjusted for age, BMI, familial history of PCAD, smoking, hypertension, diabetes, glucose, LDL-C, and Apob

Associations between serum Pcsk9 levels and SYNTAX scores were shown in Fig. 1a (r_s_ = 0.167, *P* < 0.05). The diagnostic cutoff value of PSCK9 level for medium-high SYNTAX was 122.9 ng/mL, yielding an area under the curve (AUC) of 0.63, sensitivity 81%, and specificity 40% (Supplementary Figure 1). Compared to the low SYNTAX score groups (SS≦12 and 12 < SS≦21.5, respectively), serum Pcsk9 levels were increased in the high SYNTAX score group (SS > 21.5) (*P* < 0.05, Fig. 1b). Serum Pcsk9 levels were grouped as 3 tertiles, and were then evaluated for associations between high SYNTAX scores in PMI patients. Medium (122.92≦Pcsk9 < 204.13 ng/mL) and high (Pcsk9≧204.13 ng/mL) Pcsk9 groups had greater risk of having high SYNTAX scores (SS > 21.5) than those in the low Pcsk9 group (Pcsk9 < 122.92 ng/mL), either un-adjusted or adjusted for confounding factors (all *P* < 0.05, Fig. 2).

Serum Pcsk9 was positively correlated with LDL-C levels (*r* = 0.138, *P* < 0.05), while serum Pcsk9 was not correlated with Apob levels (*r* = 0.092, *P* = 0.175) (Fig. 1c and d). After stratifying by serum LDL-C level (cutoff = 2.6 mmol/L), multivariate logistic regression analysis indicated that medium Pcsk9 levels had a greater risk of high SYNTAX scores in patients with high LDL-C levels, which were unadjusted or adjusted for confounding factors (all *P* < 0.05, Supplementary Table 1). This observation was not found in patients with low LDL-C levels after being unadjusted or adjusted for confounding factors (all *P* > 0.05, Supplementary Table 1).

### Pcsk9 level and MACE

Three hundred sixteen patients were followed up (16 patients were lost to follow-up) for the occurrence of MACE (Supplementary Table 2). After 1-year follow-up, 40 patients had occurrence of MACE, which included 3 cardiac deaths, 4 non-fatal recurrent myocardial infarctions, 26 target lesion revascularizations, 7 heart failures, and 3 strokes. Serum Pcsk9, TG, and SBP increased significantly in patients with MACE compared to levels in patients without MACE (Supplementary Table 2). After adjusting for confounding factors, univariate Cox regression analysis indicated that high levels of Pcsk9, creatinine, glucose, blood urea nitrogen (BUN) and SBP significantly associated with high risk of MACE in PMI patients (Table 3). Subsequent multivariate Cox regression analysis indicated that elevating Pcsk9 levels were associated with high risk of MACE (*P* < 0.05, Table 3). Similarly, patients with high Pcsk9 levels (≧204.13 ng/mL) had a high risk of MACE compared with those with low Pcsk9 levels (< 122.92 ng/mL) by univariate Cox regression analysis, but without statistical significance (HR = 2.28, *P* = 0.08, Table 3). Compared to patients with low serum Pcsk9 levels, patients with high serum Pcsk9 levels had lower MACE-free probability after 50-months follow-up, but without statistical significance (85% vs 65%, *P* = 0.20, Supplementary Figure 2).

**Table 3:** Univariate and Multivariate Cox regression model of patients’ characteristics and MACE events

Variables	Univariate		Multivariate	
	HR (95% CI)	***P-***value	aHR (95% CI)	***P-***value
**Age**, years	1.07 (0.98, 1.18)	0.150	–	–
**Body mass index**, kg/m^2^	1.14 (0.91, 1.42)	0.250	–	–
**SBP**, mmHg	1.02 (1.01, 1.03)	0.030*	1.01 (0.99, 1.03)	0.168
**Past history**				
Hypertension	1.15 (0.55, 2.41)	0.710	–	–
Diabetes	1.59 (0.64, 3.93)	0.320	–	–
Familial history of PCAD	1.79 (0.68, 4.71)	0.240	–	–
Smoking	2.55 (0.77, 8.44)	0.130	–	–
**Biochemistry**				
White blood cells, 10^9^/L	0.99 (0.87, 1.12)	0.890	–	–
Urea, mmol/L	1.22 (1.01, 1.48)	0.047*	1.02 (0.77, 1.35)	0.896
Lp(a), nmol/L	0.99 (0.99–1.00)	0.407	–	–
TC, mmol/L	0.97 (0.69, 1.36)	0.850	–	–
LDL-C, mmol/L	0.96 (0.67, 1.37)	0.820	–	–
LDL-C > 2.6 mmol/L	1.57 (0.59, 4.14)	0.360	–	–
VLDL-C, mmol/L	1.00 (0.65, 1.53)	0.990	–	–
Apob, g/L	1.10 (0.32, 3.83)	0.880	–	–
HDL-C, mmol/L	1.52 (0.68, 5.87)	0.130	–	–
Triglycerides, mmol/L	1.06 (0.92, 1.22)	0.450	–	–
ALT, U/L	1.00 (0.99, 1.01)	0.880	–	–
Glucose, mmol/L	1.07 (1.00, 1.13)	0.040*	0.99 (0.93, 1.08)	0.977
Creatinine, μmol/L	1.02 (1.01, 1.03)	< 0.001*	1.01 (0.98, 1.03)	0.592
**Ischemic heart disease**				
LVEF, %	0.99 (0.94, 1.04)	0.560	–	–
Creatine kinase, U/L	1.00 (1.00, 1.00)	0.950	–	–
c-Tnt, ng/Ml	1.03 (0.92, 1.16)	0.590	–	–
hs-Crp, mg/L	1.00 (0.99, 1.01)	0.910	–	–
NT-proBnp, pg/mL	1.00 (1.00, 1.00)	0.170	–	–
SYNTAX score > 21.5	1.01 (0.97, 1.06)	0.540	–	–
SYNTAX score	1.13 (0.52, 2.45)	0.750	–	–
Biomarker				
**Pcsk9, ng/mL**	1.01 (1.004, 1.011)	< 0.001*	1.01 (1.00, 1.01)	< 0.001*
**< 122.92**	Ref.	Ref.		
**122.92–204.13**	0.15 (0.57, 4.10)	0.400	–	–
**≧204.13**	2.28 (0.90, 5.82)	0.080	–	–

Variables are presented as adjusted hazard ratio (aHR), 95% confidence interval (95% CI), *P*-values were derived from univariate or multivariate analysis. *P < 0.05

*SBP* systolic blood pressure, *PCAD* premature coronary artery disease, *Lp(a)* Lipoprotein a, *TC* total cholesterol, *LDL-C* low density lipoprotein cholesterol, *Apob* apolipoprotein B, *HDL-C* high density lipoprotein cholesterol, *ALT* alanine transaminase, *LVEF* left ventricular ejection fraction, *hs-Crp* high-sensitivity C-reactive protein, *c-Tnt* cardiac Troponin T, *NT-proBnp* N-terminal pro-B type natriuretic peptide, *Pcsk9* proprotein convertase subtilisin/kexin type 9

## Discussion

CHD is a globally leading cause of death [30], however, the studies focusing on PMI are lacking. PMI accounts for about 27.7% of MI patients in China [31]. MI may be devastating, particularly in patients with young age due to its greater impact on patients’ psychological status, ability to work and the socioeconomic burden. The present study expanded previous findings and is the first study to establish a correlation of serum Pcsk9 levels and coronary artery lesions (indicated by SYNTAX scores) in PMI patients. Particularly, Pcsk9-related coronary severity may be associated with high LDL-C levels. In addition, higher serum Pcsk9 levels had an increased risk of MACE in PMI patients after PCI intervention.

The SYNTAX score is an anatomical-based tool, which are used to objectively judge the complexity of CAD and guide the decision between CABG and PCI. Using the SYNTAX score to assess coronary severity is reasonable and meets the current guidelines, such as for use in myocardial infarction [19, 32]. Recent studies have also shown that high levels of Pcsk9 can be considered as new and reliable biomarkers for the presence and severity of CHD [13, 23, 24]. Also, the circulating concentration of Pcsk9 increased in the first few hours/days after acute coronary syndrome (ACS) [23, 33]. Serum Pcsk9 levels are related to SYNTAX scores in patients with acute or stable CAD [20–23]. The correlation between serum Pcsk9 levels and the extent of disease in PMI patients has not been studied until now. The present study showed that, similar to patients with acute and stable CAD, medium and high Pcsk9 levels also had increased risk of increased coronary severity (SYNTAX score > 21.5) in PMI patients. Simultaneously, high Pcsk9 levels were associated with increased risk of MACE after PCI intervention. As SYNTAX scores increase, the incidence of adverse long-term cardiovascular events increases, including MACE [34, 35]. Therefore, results of the present study suggest thatPcsk9-related MACE maybe associated with severe coronary artery lesions in PMI patients after PCI intervention.

In the present study, in addition to Pcsk9 levels, ApoB, creatine kinase, NT-proBnp, diabetes were also independent predictors of SYNTAX scores. In addition, significant differences were found in LDL-C levels between the three SYNTAX groups. LDL-C, ApoB and SYNTAX scores were significantly positively correlated, which is also consistent with previous research [36]. However, studies have shown that hs-Crp also correlates positively with CHD severity [37], which does not agree with results in the present study for patients with PMI. According to these findings, it suggests that severe coronary lesions in various types of CAD (e.g., ST-segment elevation myocardial infarction, stable angina pectoris) may be associated with different predictors.

Hyperlipidemia is a traditional risk in all age group of CHD, which appears to be correlated with MI [38]. Particularly, familial hyperlipidemia was associated with high prevalence of MI, with a prevalence of 38% [39]. It also appears to exhibit higher concentrations of total cholesterol, triglyceride, and LDL-C in patients with MI [40, 41]. Recently, association of LDL-C with MI was found in patients aged 40 years, with an adjusted OR of 5.02 compared to controls [41]. However, the present study showed that all PMI patients did not have histories of familial hypercholesterolemia and statin use, while histories of PCAD, beta-blocker use, CCB use and ARB/ACEI use were not correlated with severity of coronary artery lesion in these patients. Although there were the associations of coronary artery lesions and LDL-C, ApoB, or lipoprotein (a) levels. Lipoprotein(a)≧60 mg/dL has been shown to be associated with MACE in patients with premature myocardial infarction [42]. However, the present study indicated that lipoprotein (a) was not associated with SYNTAX score and MACE. Furthermore, as a new risk factor for CHD, Pcsk9 could reduce liver clearance of plasma LDL-C by degrading LDL receptors [43]. Although Pcsk9 is related to the abundance of LDL receptors in liver cells, it is unclear whether the level of Pcsk9 directly reflects the activity of LDL receptors [20]. Studies have shown that Pcsk9 may speed up atherosclerosis due to promote inflammation, endothelial cell function and hypertension, and that its mechanism is not related to LDL-R [44]. Therefore, Pcsk9 may be directly related to atherosclerosis development [20, 45]. Regarding possible direct atherosclerotic effects of Pcsk9, distinguishing the roles of Pcsk9 from those of LDL-C will be difficult, because it interferes with cholesterol metabolism very rapidly by enhancing LDLR degradation at different tissue levels [23]. Interestingly, the present study showed that, after stratifying by serum LDL-C level, medium-high Pcsk9 levels were only correlated with greater risk of high SYNTAX scores in patients with high LDL-C level, in whom this observation was not found. According to these findings, Pcsk9-related severe coronary artery lesions in PMI patients did not relate to familial hyperlipidemia and may be caused by the effects of Pcsk9 on LDL receptors, resulting in increased serum LDL-C levels.

Due to the lack of clinical variables and a lack of personalized decision-making methods, the limitations of the SYNTAX score in helping decision-making between CABG and PCI have be-come obvious [34]. Combining clinical grammatical variables with clinical variables to facilitate decision-making be-tween CABG and PCI may ultimately result in improved SYNTAX scores (e.g., logistic clinical SYNTAX scores), which can objectively make tailored decisions for individual patients [34, 46]. Compared to SYNTAX score alone, the Logistic Clinical SYNTAX Score (combination of SYNTAX score, age, creatinine clearance, and LV ejection fraction) has been demonstrated to improve the prediction of mortality in subjects with complex coronary artery disease [6]. In the present study, serum Pcsk9 levels were associated with SYNTAX scores and MACE, but based on these studies, suggesting that serum Pcsk9 levels may be combined with SYNTAX scores and other variables to develop a new modified SYNTAX score to improve MACE predictions in PMI patients undergoing PCI intervention in the future.

More and more clinical applications of Pcsk9 inhibitors reported in numerous related studies show that they have a significant efficacy on reducing LDL-C and even on regression of coronary plaque. As novel lipid-lowering drugs, Pcsk9 inhibitors bind to LDL receptor on the surface of the hepatocytes, allowing more of these LDL receptors to clear LDL-C from the blood. Studies have shown that the Pcsk9 inhibitor evolocumab reduced LDL-C levels by about 60% [47]. In the GLAGOV trial [48], 64% of patients with ACS who were treated with combined statins and evolocumab achieved plaque regression after 78 weeks as examined by intravascular ultrasonography (IVUS). A recent study examined the effects of evolocumab on the extent of atherosclerotic plaque in ACS patients through serial optical coherence tomography (OCT) analysis, finding that the addition of evolocumab to statin therapy may increase fibrous cap thickness and regression of lipid-rich plaques [49], which may reduce the risk of atherosclerosis plaque ruptures. Taken together, these studies provide strong evidence supporting the current findings about associations between Pcsk9 and coronary artery lesions, providing guidance for clinical applications of Pcsk9 inhibitor.

### Study strength and limitations

The data of the present study will help to further optimize risk stratification of coronary heart disease and provide more scientific and reasonable evidence for clinical application of Pcsk9 inhibitors, especially among young people. This will also contribute to improving the prognosis of PMI patients and help to reduce the related disability and mortality. It looks forward to implementing more large-sample, multi-center prospective studies. Nevertheless, several limitations of this study must be acknowledged. First, this single-center analysis may not completely exclude the selection bias, which may partially explain some statistically insignificant correlations in this study. Therefore, it still remains necessary to perform a large-scale and prospective multi-center study to confirm the results of the present study. Second, female was excluded from this study because the number of MACE of female patients are not inadaptable to perform the analysis of regression model. Third, this is a cross-sectional study that can only establish associations, not cause-and-effect associations. Also, the MACE results were not obtained in 16 patients who either declined to answer follow-up calls or had provided incorrect telephone numbers, which may have interfered with analysis. Finally, the lack of endovascular imaging techniques that are more sensitive to detecting atherosclerosis limits exploration of this topic.

## Conclusions

Serum Pcsk9 levels are correlated to the extent of coronary artery lesions in PMI patients, especially in patients with high LDL-C. Moreover, high Pcsk9 levels are associated with risk of MACE in these patients after PCI intervention. This suggests that serum Pcsk9 may be as a biomarker for coronary artery lesion severity and may contribute to risk stratification in PMI patients, while providing guidance for clinical application of Pcsk9 inhibitors.

## Supplementary Information

**Additional file 1: Supplementary Table 1.** Multivariate logistic regression model of Pcsk9 and SYNTAX score after classifying by LDL-C levels. **Supplementary Table 2.** Baseline characteristics of patients with premature MI. **Supplementary Figure 1.** ROC curve analysis of predictive marker Pcsk9 for identifying the occurrence of high SYNTAX score. **Supplementary Figure 2.** Comparison of 1-year cardiovascular events of PMI between patients with low, medium and high Pcsk9 levels. Patients were stratified according to T1 (< 122.92 ng/mL), T2 (122.92–204.13 ng/mL), and T3 (≧204.13 ng/mL) for serum pcsk9 level.

## Data Availability

The data used to support the findings of this study are included within the article and its supplementary information files.

## Abbreviations

Pcsk9: Proprotein convertase subtilisin/kexin type 9
PMI: Premature myocardial infarction
CAG: Coronary angiography
PCI: Percutaneous coronary intervention
SYNTAX: SYNergy between percutaneous coronary intervention with [paclitaxel-eluting] TAXUS stent and cardiac surgery
LDL: Low-density lipoprotein
Apob: Apolipoprotein B
NT-proBnp: N-terminal pro-brain natriuretic peptide
CK: Creatine kinase
MACE: Major adverse cardiac events
NSTEMI: Non-ST elevation myocardial infarction
STEMI: ST-elevation myocardial infarction
CAD: Coronary artery disease
SBP: Systolic blood pressure
LVEF: Left ventricular ejection fraction (LVEF)
BUN: Blood urea nitrogen
