# Supplementary material for: Pcsk9 is associated with severity of coronary artery lesions in male patients with premature myocardial infarction
Source: Lipids Health Dis. 2021 May 27;20:56. doi: 10.1186/s12944-021-01478-w (PMC8161665; doi:10.1186/s12944-021-01478-w)
Supplement: Supplementary file 1 — Additional file 1: Supplementary Table 1. Multivariate logistic regression model of Pcsk9 and SYNTAX score after classifying by LDL-C levels. Supplementary Table 2. Baseline characteristics of patients with premature MI. Supplementary Figure 1. ROC curve analysis of predictive marker Pcsk9 for identifying the occurrence of high SYNTAX score. Supplementary Figure 2. Comparison of 1-year cardiovascular events of PMI between patients with low, medium and high Pcsk9 levels. Patients were stratified according to T1 (< 122.92 ng/mL), T2 (122.92–204.13 ng/mL), and T3 (≧204.13 ng/mL) for serum pcsk9 level. [file 12944_2021_1478_MOESM1_ESM.docx]

**Pcsk9 is associated with severity of coronary artery lesions in male patients with premature myocardial infarction**

**Supplementary Table 1. Multivariate logistic regression model of Pcsk9 and SYNTAX score after classifying by LDL-C levels.**

| **LDL-C subgroup** | **Model** | **aOR (95%CI)** | ***P*-value** |
| --- | --- | --- | --- |
| **>2.6 mmol/L** |  |  |  |
|  | Unadjusted | 1.81(1.19, 2.75) | 0.006* |
|  | Model 1 | 1.89(1.14, 3.15) | 0.027* |
|  | Model 2 | 1.90(1.11, 3.27) | 0.020* |
|  | Model 3 | 2.12(1.19, 3.75) | 0.010* |
| **≤2.6 mmol/L** |  |  |  |
|  | Unadjusted | 1.42(0.56, 3.56) | 0.461 |
|  | Model 1 | 0.87(0.23, 3.36) | 0.844 |
|  | Model 2 | 0.95(0.16, 5.74) | 0.957 |
|  | Model 3 | 1.27(0.15, 11.21) | 0.828 |

Multivariate logistic model 1: adjustment for age and body mass index (BMI).

Multivariate logistic model 2: adjustment for age, BMI, SBP, history of hypertension, diabetes mellitus, familial PCAD, smoking.

Multivariate logistic model 3 = adjustment for age, BMI, SBP, history of hypertension, diabetes mellitus, familial PCAD, smoking, triglycerides, creatinine, SYNTAX score, Apob, glucose.

Pcsk9, proprotein convertase subtilisin/kexin type 9; LDL-C, low density lipoprotein cholesterol; aOR, adjusted odds ratio; 95% CI, 95% confidence interval. **P*<0.05

**Supplementary Table 2. Baseline characteristics of patients with premature MI.**

| **Variables** | **Maces (+)**  **(=40)** | **Maces (-)**  **(n=276)** | ***P*-value** |
| --- | --- | --- | --- |
| **Age**, years | 42.50(7.00) | 40.00(7.00) | 0.10 |
| **Body mass index**, kg/m^2^ | 25.40(3.65) | 24.80(3.10) | 0.34 |
| **SBP**, mmHg | 141.04(20.77) | 131.23(23.33) | 0.04* |
| **Past history** |  |  |  |
| Hypertension | 20(50.00) | 121(43.84) | 0.54 |
| Diabetes | 9(22.50) | 35(12.68) | 0.24 |
| Familial history of PCAD | 7(17.50) | 25(9.06) | 0.18 |
| Smoking | 36(90.00) | 208(75.36) | 0.10 |
| **Biochemistry** |  |  |  |
| White blood cells, 10^9^/L | 11.15(3.35) | 10.83(3.80) | 0.87 |
| Urea, mmol/L | 4.70(2.70) | 4.30(1.90) | 0.19 |
| TC, mmol/L | 4.99(0.84) | 4.95(1.38) | 0.60 |
| LDL-C, mmol/L | 3.32(0.65) | 3.33(1.03) | 0.81 |
| LDL-C >2.6 mmol/L | 33(82.50) | 207(75.00) | 0.41 |
| VLDL-C, mmol/L | 0.55(0.56) | 0.48(0.49) | 0.46 |
| Apob, g/L | 1.21(0.25) | 1.18(0.42) | 0.78 |
| HDL-C, mmol/L | 0.93(0.23) | 0.91(0.26) | 0.45 |
| Triglycerides, mmol/L | 2.33(1.78) | 2.09(1.42) | 0.03* |
| Alt, U/L | 47.40(58.30) | 54.30(38.70) | 0.72 |
| Glucose, mmol/ L | 6.09(3.50) | 5.62(2.62) | 0.36 |
| Creatinine, μmol/L | 81.50(24.00) | 76.00(18.00) | 0.08 |
| **Ischemic heart disease** |  |  |  |
| LVEF, % | 50(11.00) | 50(11.00) | 0.70 |
| Creatine kinase, U/L | 1558.00(2934.50) | 1692.00(2142.00) | 0.79 |
| c-Tnt, ng/mL | 3.74(5.54) | 3.46(4.90) | 0.76 |
| hs-Crp, mg/L | 4.54(10.33) | 6.54(13.01) | 0.31 |
| NT-proBnp, pg/mL | 516.50(753.05) | 432.85(841.55) | 0.45 |
| **SYNTAX score** | 20.25(10.25) | 17.00(13.00) | 0.23 |
| **SYNTAX score >21.5** | 14(35.00) | 86(31.16) | 0.63 |
| **Biomarker** |  |  |  |
| Pcsk9, ng/mL | 208.99(126.80) | 152.76(111.26) | <0.01* |

Variables are presented as mean (SD), median (IQR), or n (%), p values were derived from one-way analysis of variance, the Kruskal-Wallis test or χ2 test. **P*<0.05

SBP, systolic blood pressure; PCAD, premature coronary artery disease; Lp(a), Lipoprotein a; TC, total cholesterol; LDL-C, low density lipoprotein cholesterol; Apob, apolipoprotein B; HDL-C, high density lipoprotein cholesterol; Alt, alanine transaminase; LVEF, left ventricular ejection fraction; hs-Crp, high-sensitivity C-reactive protein; c-Tnt, cardiac Troponin T; NT-proBnp, N-terminal pro-B type natriuretic peptide; Pcsk9, proprotein convertase subtilisin/kexin type 9.

**Supplementary Fig. 1**


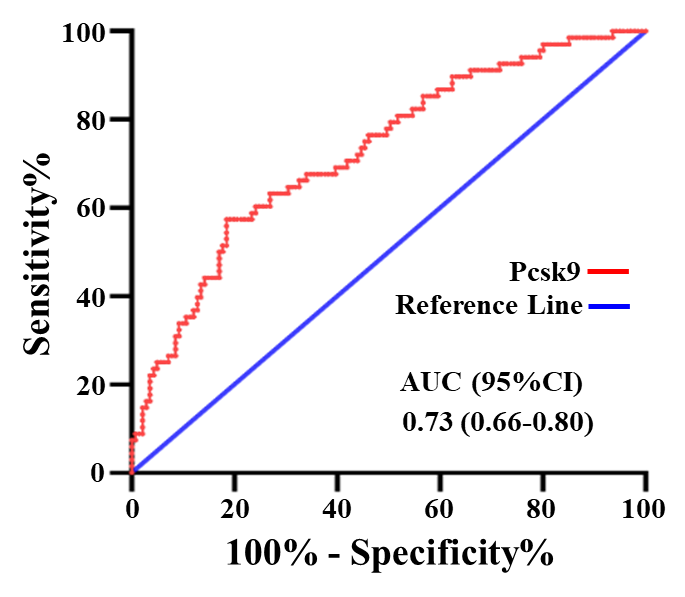


**Supplementary Fig. 1 ROC curve analysis of predictive marker Pcsk9 for identifying the occurrence of high SYNTAX score.**

**Supplementary Fig. 2**


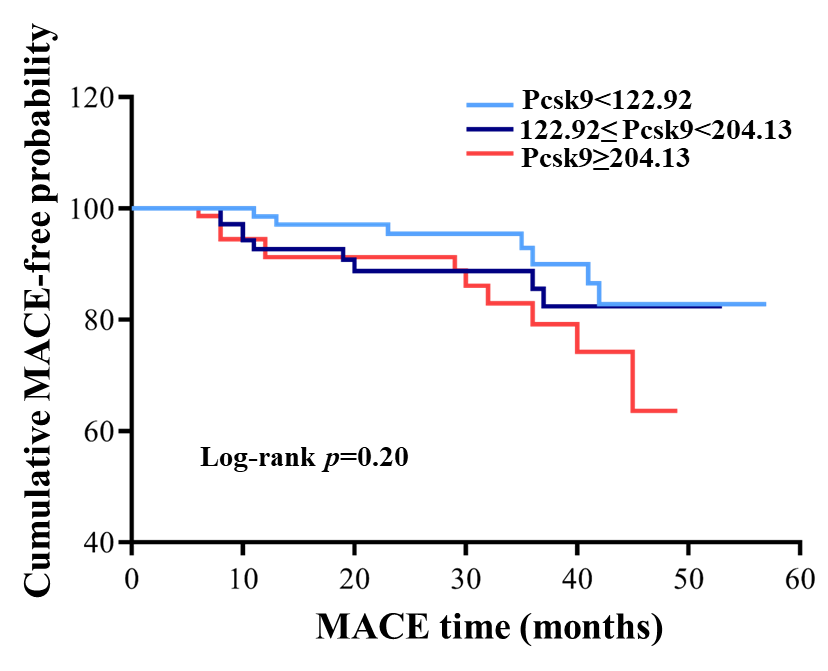


**Supplementary Fig. 2 Comparison of 1-year cardiovascular events of PMI between patients with low, medium and high Pcsk9 levels.** Patients were stratified according to T1 (<122.92 ng/mL), T2 (122.92-204.13 ng/mL), and T3 (≧204.13 ng/mL) for serum pcsk9 level.
